# Women’s exposure to commercial milk formula marketing: a WHO multi-country market research study

**DOI:** 10.1186/s12992-024-01088-y

**Published:** 2024-11-28

**Authors:** Christiane Horwood, Sphindile Mapumulo, Lyn Haskins, Tanya Doherty, Gillian Kingston, Nigel Rollins, the World Health Organization (WHO) multi-country study group

**Affiliations:** 1 Centre for Rural Health, University of KwaZulu-Natal, Durban, South Africa; 2 Health Systems Research Unit, South African Medical Research Council, Capetown, South Africa; 3 Kings Business School, Kings College, London, England; 4 Department of Maternal, Newborn, Child and Adolescent Health and Ageing, World Health Organization, Geneva, Switzerland

**Keywords:** Infant feeding, Commercial milk formula, Marketing, Child health, International code of marketing of breastmilk substitutes

## Abstract

**Background:**

Marketing of commercial milk formula (CMF) is well resourced and has influenced societal beliefs and practices that have undermined breastfeeding. This has occurred despite legislation in many countries largely reflecting the provisions of the International Code of Marketing of Breast-milk Substitutes.

**Methods:**

A cross-sectional survey was conducted in seven countries: Bangladesh, Mexico, Morocco, Nigeria, South Africa, United Kingdom and Viet Nam to explore the scope and nature of CMF marketing among pregnant women and mothers. A marketing-research methodology was adopted using convenience sampling of women stratified according to infant feeding practices and the infant’s age.

Participants were identified in hospitals and clinics, as well as in the street, markets and shopping malls. In each country the sample size comprised 300 pregnant women, 150 mothers of children aged > 18 months who were breastfeeding without giving CMF and 600 women feeding their children with CMF. Data were collected using a questionnaire administered on tablets by trained field workers.

**Results:**

Interviews were conducted with 8528 women between October 2019 and March 2021. Overall, 3095/7480 (41.3%) of women reported exposure to CMF marketing ranging from 3% in Morocco to 92% in Viet Nam. The commonest marketing site in all countries was television, but advertising in-store and in magazines and newspapers was also common. In most countries, CMF advertising on social media, websites and YouTube was less compared to traditional media. Reports of receiving free CMF samples varied from 3.1% in Nigeria to 34.6% in Viet Nam. Health professionals were the most common source of advice to mothers about starting CMF and which CMF brand to use.

**Conclusions:**

The study provides quantitative data about CMF marketing and insights on how marketing companies develop effective messages, helping to explain how individual vulnerabilities or aspirations are integrated into marketing strategies. The findings reaffirm the need for action across political and health domains to counter actions of CMF companies. This will require effective national legislation fully reflecting the Code and action by professional bodies to protect health professionals from targeting by CMF marketing. Marketing-research methods could be employed to develop messaging in support of breastfeeding and breastfeeding-friendly policies.

**Supplementary Information:**

The online version contains supplementary material available at 10.1186/s12992-024-01088-y.

## Introduction

The marketing of commercial milk formula (CMF), also known as breastmilk substitutes (BMS), and how it influences individual and societal beliefs, values and practices has been described by public health researchers and child health advocates [1, 2]. However, these reports have generally focussed on single geographies, specific strategies such as health professional sponsorship, health claims on product packaging or advertising in the media [3–5]. Despite the decision by the World Health Assembly to develop and adopt the International Code of Marketing of Breast-milk Substitutes in 1981 and agree to subsequent resolutions (hereafter jointly referred to as the Code) [6], the CMF industry continues to actively market CMF products [7, 8].

The scope of marketing has recently been more fully characterised [9]. Here, we define marketing as “any form of commercial communication or activity that is “designed to, or has the effect of, increasing recognition, appeal and [or] consumption of particular products and services” but this excludes transportation and sales of products [10]. In business, marketing is considered “a strategic approach … focused on maximising sales and shareholder returns” [11]. While all sections of society are targets of marketing strategies, individual mothers, families and health professionals represent particular high value for the CMF industry. Firstly, because of the purchasing power of families, and secondly, because of the respected position of health professionals in society and their influence on infant feeding decision-making [7, 12].

CMF marketing is extremely well-resourced, with total spending in excess of US$ 3.5 billion annually, increasing year-on-year in many low- and middle-income countries [7, 9, 13]. Marketing strategies are carefully designed, researched and tested prior to deployment, especially among those who will be trend-setters and influence normative practices. CMF marketing starts with developing an understanding of the lived experiences of the customer [7]. Market research is used to identify “pain points”, vulnerabilities in life that can be leveraged to present products as “solutions” to life’s complexities; it seeks to identify the “highest order emotional benefit” such as an aspiration or uncertainty that can be used to build brand relevance and credibility [7, 14]. It examines the appeal of messaging and design of packaging to determine product acceptability and to find “category entry points”, i.e. mental cues that customers use to access thoughts and memories when in a buying situation [15].

Therefore, while much of public health research focusses on describing practices, assessing interventions and impact, market research examines principally examines behaviours and how these might be influenced. Methods are similarly robust but have different objectives and different sampling frames. Market research methodologies recognize that not everyone is a consumer, and not all consumers are the same. Consumers have different social and financial requirements and aspirations when choosing a product, and targeted messages are used to influence the practices of different types of consumers, especially those who are deemed to be important or influential. The increase in CMF sales demonstrates how these strategies have been very effective in influencing social norms and normalizing the use of CMF in many settings resulting in unhealthy infant feeding practices and adverse public health outcomes [15, 16].

Here, we present the quantitative results from a large study conducted in seven countries exploring women’s, families and health professional exposures to CMF marketing, and women’s sources of information and advice about infant feeding. Throughout, we use the term CMF rather than BMS even though they refer to the same range of products. First, to highlight the artificial and ultra-processed nature of CMF products; and second, because, the term ‘substitute’ conveys the notion of equivalence – which high quality evidence has demonstrated to be untrue [17].

## Methods

### Study design

The World Health Organization (WHO) commissioned M&C Saatchi World Services, a specialist communication and research unit within M&C Saatchi, to design and implement a multi-country cross-sectional study. Local academic partners were identified in each participating country to provide expert guidance and oversight during implementation of the study, including study design, ethics approval, piloting, adaptation of tools and data collection. Further, the study was overseen by an International Scientific Advisory Group composed of global research leaders in the field of child health and nutrition, epidemiology and social marketing. Consumer-focussed sampling, data collection and analysis frameworks were adopted as if to inform a commercial marketing initiative i.e. information to understand target markets and to develop effective strategies to promote products.

The study aimed to characterise the scope and nature of marketing of CMF products among pregnant women, mothers, health professionals and other key stakeholders in urban settings in seven countries: Bangladesh, Mexico, Morocco, Nigeria, South Africa, United Kingdom and Viet Nam. Here, we report quantitative findings from data collected among pregnant women and mothers.

### Study setting and population

The countries were purposively selected to include representation from all six WHO regions. Two large cities were purposively selected in each participating country (Appendix 1) because as major urban centres they were considered to be progressive and influential in setting societal trends and behaviours. Such approaches are taken in market research to inform marketing campaigns. The data are therefore not intended to be representative of the general population of the participating country.

### Sampling

Recruitment methods were tailored for each country. Participants were selected using stratified sampling according to their socioeconomic status and current feeding practice, using convenience sampling techniques. The same stratification criteria: pre- or post-natal mothers, infant feeding practice, infant age and SES, were applied in each country to ensure equal representation as shown in Appendix 2. Non-probability techniques were used to ensure that all sub-categories of participants were represented, according to the feeding practice and the age of the children. Mothers with children aged between 0–18 months of age and pregnant women were selected to ensure representation of two feeding practices: breastfeeding only and those who used CMF, either exclusively or in a mixed feeding regimen. In each country the targeted sample comprised 300 pregnant women, 150 mothers who were breastfeeding without giving CMF (infants aged 0–12 months) and 600 women who were feeding their children with CMF (Age 0-3months: 75; 4-6months: 75; 7-12months: 75; 13-18months: 75). Research sites were selected by identifying localities that were representative of different socioeconomic status (SES) groups. To strengthen the representativity of the survey, quotas by SES were applied, and participants were assigned to low, medium, and high SES groups according to locally developed criteria. The sample size by population, SES group and setting is shown in Appendix 2.

Pregnant women and mothers with infants were screened and recruited by trained field workers in hospitals and clinics with permission from the responsible authority. In addition, as in market research, participants were recruited online, door-to-door, and through street-based recruitment, undertaken in areas close to hospital or clinics, and also in markets, shopping malls and on-the-street.

The COVID-19 pandemic occurred during data collection. Data collection was temporarily stopped, and when resuming the recruitment, methods were adapted according to national COVID-19 regulations in each country as these evolved during the course of the study. This included online recruitment when in-person data collection was not possible. All field workers received training in COVID-19 safety protocols and were provided with protective equipment. During the pandemic field workers and participants were required to complete a health screen confirming that they were not experiencing any COVID-19 symptoms. Adherence to protocols was monitored by the research team.

### Data collection

A survey questionnaire was developed and first piloted in Viet Nam (Appendix 3). In each country, the questionnaire was also adapted to align with the local context and pre-tested to check that questions were well understood.

As part of the adaptation of the master data collection tools in each country, desk reviews and marketing analyses were conducted to better characterise the study context. These analyses informed the themes explored within the survey instruments and ensured tools were appropriate for each country. Desk reviews and marketing analyses included mapping of infant CMF sales and market size; documenting local advertising landscapes to analyse and interpret media content; content analysis from news, blogs, and specialist sites to understand consumption of online and offline content; social media content of infant feeding and CMF narratives. This preliminary work examined how populations are exposed to media and how they engage with the wider topic of infant feeding.

Data were collected between October 2019 and March 2021. The survey questionnaire was administered using face-to-face interviews and captured directly onto tablets using computer-assisted personal interview (CAPI) assisted software. Interviews were conducted in the local language as follows: in Bangladesh the language was Bengali; in Mexico the language was Spanish; in Morocco Arabic or French were used; Nigeria, South Africa and the United Kingdom the language was English; and in Viet Nam Vietnamese was used.

Fieldworkers were trained to administer the screening tool and questionnaire by M&C Saatchi World Services staff and local partners. Adherence to protocols, interviewer performance and quality of data was monitored by local quality assurance managers and academic partners.

### Socioeconomic status

Local academic partners advised on the appropriate and commonly used measures of SES in each country of study including income, household assets, occupation of main income provider, household size and occupation. The measurement of SES in each country was guided by academic experts and discussion with the local M&C Saatchi team or research agency resulting in setting-specific methods of determining SES. In Bangladesh and Morocco, SES was determined from net household monthly income after tax and grouped into low, medium, or high SES groups. In Nigeria, South Africa and Viet Nam, monthly household income was combined with household assets suited to the local context, to create low, medium and high SES groups. A list of assets used is given in Appendix 4. In the United Kingdom (UK), income and occupation of the main income earner were combined and categorised into low, medium and high SES groups; and in Mexico, questions about education, household size and assets, and the number of people employed in the house were grouped to determine different levels of SES.

### Data analysis

All data were checked and cleaned prior to analyses. Data were analysed based on data collection tools, code books and datasets from each participating country using SPSS v.28. Data presented are descriptive using simple frequencies and percentages.

### Oversight and ethical considerations

The research was overseen by an International Scientific advisory group of expert scientists. Ethical approval for the study was obtained from the World Health Organization Research Ethics Committee in August 2019 (ERC 003235). Each participating country obtained ethical approval from the relevant ethics committees. Informed consent was obtained from all participants. Confidentiality was assured and participants were able to withdraw at any stage of the study.

## Results

There were 1,050 survey participants in five countries, 1,178 participants in Bangladesh and 1,052 participants in the UK, giving a total of 8,528 participants. Sociodemographic information about survey participants is shown in Table 1.

**Table 1:** Characteristics of participants

	**Bangladesh** *N*=1178 n (%)	**Mexico** *N*=1050 n (%)	**Morocco** *N*=1050 n (%)	**Nigeria** *N*=1050 n (%)	**South** **Africa** *N*=1050 n (%)	**United Kingdom** *N*=1052 n (%)	**Viet Nam** *N*=1050 n (%)
**Mean age** **(range)**	25 (18–50)	27 (18–50)	30 (18–50)	31 (18–46)	NA^1^	33 (18–47)	NA^1^
**Age group**							
18–24	616 (52)	397 (38)	174 (17)	136 (13.0)	295 (28)	81 (8)	138 (13)
25–29	352 (30)	280 (27)	314 (30)	352 (33.5)	295 (28)	206 (20)	387 (37)
30–34	155 (13)	201 (19)	321 (31)	321 (30.6)	234 (22)	407 (39)	317 (30)
35–39	52 (4)	139 (13)	132 (13)	192 (18.3)	173 (17)	282 (27)	160 (15)
40–44	3 (0)	29 (3)	99 (9)	42 (4.00)	52 (5)	70 (7)	40 (4)
45–50	0 (0)	4 (0)	10 (1)	7 (0.7)	1 (0)	6 (1)	8 (1)
**Socioeconomic Status**							
Low	393 (33.4)	350 (33.3)	350 (33.3)	350 (33.3)	349 (33.2)	330 (31)	351 (33)
Medium	393 (33.4)	350 (33.3)	350 (33.3)	349 (33.2)	351 (33.4)	359 (34)	354 (34)
high	392 (33.3)	350 (33.3)	350 (33.3)	351 (33.5)	350 (33.3)	363 (35)	345 (33)
**Pregnant or postnatal**							
Pregnant	270 (23)	302 (29)	301 (28.7)	313 (29.9)	300 (28.6)	300(28.5)	301 (28.7)
Postnatal	908 (77)	754 (71.8)	755 (71.9)	750 (71.4)	765 (72.9)	772 (73.4)	749 (71.3)
**Education**							
No education	36 (3)	4 (0)	0 (0)	301 (29)	0 (0)	1 (0)	0 (0)
Primary education	543 (46)	102 (10)	246 (24)	0 (0)	44 (4)	2 (0)	14 (1)
Secondary education	427 (36)	818 (78)	517 (49)	42 (4)	725 (70)	277 (26)	475 (36)
Higher education	131 (11)	126 (12)	272 (26)	460 (44)	266 (26)	549 (52)	552 (53)
Post Graduate	41 (4)	0 (0)	14 (1)	246 (24)	0(0)	213 (20)	0 (0)
**Current work situation**							
Full time (≥30 hours per week)	66 (5.6)	120(11.4)	9 (0.9)	460 (43.8)	442 (42.1)	231 (22.0)	382 (36.4)
Part time (8–29 hours per week)	29 (2.5)	130 (12.4)	51 (4.9)	199 (19.0)	92 (8.8)	168 (16.0)	100 (9.5)
Part time (<8 hours per week)	9 (0.8)	53 (5.0)	41 (3.9)	109 (10.4)	26 (2.5)	11 (1.0)	17 (1.6)
Housewife / full time mother	1029 (87.4)	720 (68.6)	887 (84.5)	63 (6.0)	143 (13.6)	129 (12.3)	304 (29.0)
On maternity leave	20 (1.7)	4 (0.4)	47 (4.5)	87 (8.3)	60 (5.7)	377 (35.8)	232 (22.1)
Unemployed / looking for a job	2 (0.2)	14 (1.3)	11 (1.0)	119 (11.3)	232 (22.1)	122 (11.6)	14 (1.3)
In full time education	23 (2.0)	9 (0.9)	4 (0.4)	13 (1.2)	55 (5.2)	14 (1.3)	1 (0.1)

^1^data not collected

### Exposure to CMF marketing

Overall, the proportion of mothers who had been exposed to CMF marketing was 41.3% (3095/7480), and varied widely between participants in different settings, ranging from 3% in Morocco to 84% and 92% in the United Kingdom and Viet Nam respectively (Table 2). Among respondents who reported exposure to marketing, the most common site for marketing in all participating countries was television. Traditional marketing sites, including in-store advertising and advertising in magazines and newspapers, remained frequent in many countries. Reports of CMF advertising on social media, websites and YouTube were lower in most countries when compared to more traditional media sites. However, exceptions were the high income setting of the United Kingdom, where 50% of participants reported seeing CMF advertising on social media, and in Morocco, where overall exposure to advertising was lowest, social media and online media were the most frequently reported advertising sites. CMF advertising in health centres or hospitals were reported in all countries with the exception of Morocco.

**Table 2:** Self-reported exposure to marketing of CMF products in the preceding 12 months

	**Bangladesh** ***N*=1178** n (%)	**Mexico** ***N*=1050** n (%)	**Morocco** ***N*=1050** n (%)	**Nigeria** ***N*=1050** n (%)	**South Africa** ***N*=1050** n (%)	**United Kingdom** ***N*=1052** n (%)	**Viet Nam** ***N*=1050** n (%)
Proportion of women reporting exposure to CMF marketing in the preceding 12 months	321 (27%)	413 (39%)	27 (3%)	254 (24%)	222 (21%)	888 (84%)	970 (92)
**Locations where women reported frequently seeing any type of CMF marketing**							
	***N*=321** n (%)	***N*=413** n (%)	***N*=27** n (%)	***N*=254** n (%)	***N*= 222** n (%)	***N*=888** n (%)	***N*=970**^**1**^
TV (incl cable TV)	253 (78.8)	338 (81.8)	7 (25.9)	193 (76)	154 (69.4)	672 (75.7)	
Radio	1 (0.3)	16 (3.9)	0	15 (5.9)	9 (4.1)	18 (2.0)	
YouTube	67 (20.9)	93 (22.5)	11 (40.7)	2 (0.8)	5 (2.3)	180 (20.3)	
Any website ^2^	4 (1.2)	42 (10.2)	2 (7.4)	1 (0.4)	6 (2.7)	168 (18.9)	
Social media /Facebook	50 (15.6)	57 (13.8)	19 (70.4)	21 (8.3)	18 (8.1)	444 (50.0)	
Online discussion or chat forum	No variable	2 (0.5)	0	2 (0.8)	0	127 (14.3)	
Mothers club or online group	No variable	9 (2.2)	No variable	No variable	2 (0.9)	166 (18.7)	
In an email	0	0	0	1 (0.4)	1 (0.5)	134 (15.1)	
In the post	3 (0.9)	2 (0.5)	0	10 (3.9)	2 (0.9)	74 (8.3)	
In a health centre, hospital or clinic	39 (12.1)	61 (14.8)	0	35 (13.8)	35 (15.8)	124 (14.0)	
Magazine or newspaper	8 (2.5)	73 (17.7)	0	5 (2.0)	40 (18.0)	203 (22.9)	
Billboard	15 (4.7)	38 (9.2)	0	28 (11.0)	15 (6.8)	39 (4.4)	
Supermarket/shop/ market (in store)	53 (16.5)	83 (20.1)	0	31 (12.2)	62 (27.9)	306 (34.5)	
Supermarket/shop (online)	35 (10.9)	23 (5.6)	0	19 (7.5)	20 (9.0)	141 (15.9)	
Other	2 (0.6)	1 (0.2)	0	0	0	32 (3.6)	

^1^No data collected in Vietnam

^2^includes variables ‘company website’, ‘professional or expert website’ and ‘any website’

All participants were asked about the marketing approaches they had experienced. These included exposure to unsolicited contact from a CMF company, for example invitations to participate in commercially sponsored baby clubs or baby helplines, or receiving direct private messages from CMF companies (Table 3). This type of direct contact with women was uncommon in most participating countries but was reported frequently in the UK (36.4%) and Viet Nam (40.8%). Promotions offering discounts on the price of CMF or free gifts were widespread, and most common in the UK (16.5%) and Viet Nam (25.4%). In Morocco, where reports of marketing exposure were low, reports of unsolicited approaches by CMF companies were quite frequent. Reports of receiving free CMF samples varied widely between countries from 3.1% in Nigeria to 26.5% and 34.6% in Morocco and Viet Nam respectively. Very few participants reported approaching CMF companies themselves, for example accessing information on social media, participating in competitions linked to advertising or signing up for baby clubs sponsored by companies.

**Table 3:** Reported exposure to company-initiated and consumer-initiated marketing strategies

**ALL MOTHERS**	**Bangladesh** ***N*=1178**	**Mexico** ***N*=1050**	**Morocco** ***N*=1050**	**Nigeria** ***N*=1050**	**South Africa** ***N*=1050**	**United Kingdom** ***N*=1050**	**Viet Nam** ***N*=1050**
**Mothers who reported unsolicited contact with CMF companies**							
Any unsolicited information or contact from a formula company that you have *not requested* ^1^	7 (0.6)	127 (12.1)	100 (9.5)	8 (0.8)	92 (8.8)	383 (36.4)	428 (40.8)
Promotion for discount on commercial milk formula	61 (5.2)	72 (6.9)	128 (12.2)	16 (1.5)	78 (7.4)	279 (26.5)	386 (36.8)
Free bottles or teats	No variable	66 (6.3)	125 (11.9)	12 (1.1)	46 (4.4)	263 (25.0)	77 (7.3)
Gifts from formula companies e.g. toy, bag or clothing	34 (2.9)	86 (8.2)	132 (12.6)	16 (1.5)	37 (3.5)	174 (16.5)	267 (25.4)
An invitation to a competition from a formula company or from a shop	5 (0.4)	26 (2.5)	128 (12.2)	7 (0.7)	44 (4.2)	141 (13.4)	84 (8.0)
An invitation from a formula company to participate in a research survey or interview	8 (0.7)	13 (1.2)	144 (13.7)	10 (1.0)	26 (2.5)	54 (5.1)	104 (9.9)
Pop up/seen advert on social media	98 (8.3)	127 (12.1)	117 (11.1)	28 (2.7)	63 (6.0)	489 (46.5)	474 (45.1)
**Mothers who reported receiving free samples of CMF**							
In hospital	34 (2.9)	164 (15.6)	199 (19.0)	25 (2.4)	86 (8.2)	180 (17.1)	299 (28.5)
Outside hospital	37 (3.1)	47 (4.5)	207 (19.7)	18 (1.7)	46 (4.4)	39 (3.7)	226 (21.5)
Total ^2^	55 (4.7)	186 (17.7)	287 (26.5)	33 (3.1)	110 (10.5)	207 (19.7)	363 (34.6)
**Mothers who reported initiating contact with CMF companies**							
Followed a formula company on social media	18 (1.5)	50 (4.8)	75 (7.1)	8 (0.8)	30 (2.9)	120 (11.4)	180 (17.1)
Used any information from formula company’s website	18 (1.5)	21 (2.0)	81 (7.7)	5 (0.5)	36 (3.4)	239 (22.7)	94 (9.0)
Participate in baby competitions run by formula companies	2 (0.2)	4 (0.4)	57 (5.4)	3 (0.3)	26 (2.5)	79 (7.5)	19 (1.8)
Registered for updates/newsletter from formula companies	No variable	20 (1.9)	No variable	No variable	14 (1.3)	167 (15.9)	78 (7.4)
Signed up/registered for a baby club run by a formula company	No variable	16 (1.5)	No variable	No variable	20 (1.9)	233 (22.1)	20 (1.9)
Followed a person on Instagram/Facebook to obtain information about formula feeding	28 (2.4)	61 (5.8)	83 (7.9)	10 (1.0)	29 (2.8)	84 (8.0)	No variable
None of the above	1126 (95.6)	893 (85.0)	775 (73.8)	1026 (97.7)	937 (89.2)	569 (54.1)	803 6.5

^1^Includes having received ‘any unsolicited information or contact from a formula company by email, post, phone or text message’ AND ‘an invite to join a baby club run by a formula company’ AND ‘information about a helpline run by a formula company’ AND ‘received private messages from formula companies on Facebook or other social media’

^2^Received free sample of formula milk either in the hospital, outside the hospital, or both

#### Sources of advice about CMF feeding

Participants were asked who had advised them to start feeding their baby with CMF (Table 4). Although family members and friends were often reported as giving this type of advice, health professionals including doctors, nurses, paediatricians, midwives and pharmacists were the most commonly reported source of advice. This was similar across all settings.

**Table 4:** Persons women reported to have advised them to give CMF products

**ALL MOTHERS**	**Bangladesh** ***N*=1178**	**Mexico** ***N*=1050**	**Morocco** ***N*=1050**	**Nigeria** ***N*=1050**	**South Africa** ***N*=1050**	**United Kingdom** ***N*=1052**	**Viet Nam** ***N*=1050**
**Individuals reported by participants to have advised them to feed with CMF**							
Spouse/partner/husband	268 (22.8)	106 (10.1)	146 (13.9)	105 (10.0)	164 (15.6)	187 (17.8)	224 (21.3)
Mother/mother in law	237 (20.1)	282 (26.9)	150 (14.3)	234 (22.3)	282 (26.9)	283 (26.9)	432 (41.1)
Other family members	209 (17.7)	187 (17.8)	164 (15.6)	121 (11.5)	171 (16.3)	171 (16.3)	498 (47.4)
Close friends	121 (10.3)	164 (15.6)	191 (18.2)	225 (21.4)	245 (23.3)	240 (22.8)	628 (59.8)
People in my community	56 (4.8)	40 (3.8)	41 (3.9)	24 (2.3)	75 (7.1)	47 (4.5)	124 (11.8)
People on social media/chat forums	09 (0.8)	12 (1.1)	98 (9.3)	28 (2.7)	34 (3.2)	55 (5.2)	136 (13.0)
Phone help line	02 (0.2)	0	43 (4.1)	04 (0.4)	01 (0.1)	04 (0.4)	No variable
People I watch or follow on TV/radio/social media	64 (5.4)	09 (0.9)	125 (11.9)	21 (2.0)	17 (1.6)	24 (2.3)	68 (6.5)
CMF company rep/sales person	02 (0.2)	40 (3.8)	13 (1.2)	08 (0.8)	37 (3.5)	07 (0.7)	264 (25.1)
Health professional ^1^	546 (46.3)	360 (34.3)	339 (32.3)	454 (43.2)	192 (18.3)	272 (25.9)	307 (29.2)
Dietician or nutritionist	09 (0.8)	32 (3.0)	24 (2.3)	66 (6.3)	11 (1.0)	24 (2.3)	No variable
Antenatal class	05 (0.4)	05 (0.5)	09 (0.9)	131 (12.5)	49 (4.7)	31 (2.9)	43 (4.1)
None of these	431 (36.6)	317 (30.2)	145 (13.8)	227 (21.6)	331 (31.5)	420 (39.9)	103 (9.8)
Other	16 (1.4)	07 (0.7)	16 (1.5)	14 (1.3)	06 (0.6)	01 (0.1)	59 (5.6)

^1^includes general practitioner, specialist doctor, pediatrician, nurse, midwife, or pharmacist

### Choice of CMF brand

All participants who reported they had ever fed a baby with CMF (either this child or a previous child) or were planning to formula feed if they were pregnant, were shown a wide variety of CMF products (based on common CMF products available in each country). Participants were asked whether they had heard of each product and then asked which of these products they perceived to be the best and the reason for this. Friends and family were commonly reported as being influential in mother’s decision-making about which CMF brand to use. Health professionals were also important influencers, particularly in Mexico (53.2%) and Bangladesh (43.1%). Mothers perceptions about the quality of the CMF brand were also important, for example perceived equivalence to breastmilk was the reason given by 45% of women in South Africa and 51% of women in Viet Nam for their choice of CMF brand (Table 5).

**Table 5:** Reported reasons for participants’ choice of CMF brand among women who had ever formula fed or planned to formula feed

**Women who had previously formula fed or were planning to do so**	Bangladesh ***N*=655**	Mexico ***N*=667**	Morocco ***N*=604**	Nigeria ***N*=600**	South Africa ***N*=884**	United Kingdom ***N*=602**	Viet Nam ***N*=674**
I was given this brand in hospital/health clinic	136 (20.8)	241 (36.1)	144 (23.8)	24 (4.0)	60 (6.8)	135 (22.3)	30 (4.5)
It was recommended to me by a health professional	282 (43.1)	355 (53.2)	72 (11.9)	155 (25.8)	109 (12.3)	111 (18.4)	81 (12.0)
I was given a free sample outside of the hospital /health clinic	19 (2.9)	58 (8.7)	72 (11.9)	12 (2.0)	44 (5.0)	10 (1.7)	29 (4.3)
I received a promotion	4 (0.6)	18 (2.7)	14 (2.3)	3 (0.5)	20 (2.3)	12 (2.0)	19 (2.8)
It was the one I could afford	109 (16.6)	46 (6.9)	45 (7.5)	55 (9.2)	117 (13.2)	109 (18.1)	176 (26.1)
My friends and/or family use this brand	210 (32.1)	165 (24.7)	179 (29.6)	284 (47.3)	341 (38.6)	289 (48.0)	280 (41.5)
I used this brand before	76 (11.6)	77 (11.5)	81 (13.4)	110 (18.3)	137 (15.5)	151 (25.1)	148 (22.0)
I saw an advert for it e.g. in a magazine or on the television	61 (9.3)	19 (2.8)	55 (9.1)	27 (4.5)	35 (4.0)	103 (17.1)	148 (22.0)
It was discussed on social media	10 (1.5)	1 (0.1)	60 (9.9)	18 (3.0)	32 (3.6)	74 (12.3)	113 (16.8)
It was the only one available	No variable	10 (1.5)	No variable	No variable	39 (4.4)	10 (1.7)	48 (7.1)
It is most suitable for the baby’s condition	232 (35.4)	77 (11.5)	114 (18.9)	75 (12.5)	94 (10.6)	94 (16.1)	333 (49.4)
It was prescribed for my baby	No variable	No variable	No variable	No variable	No variable	25 (4.2)	No variable
I haven’t decided which formula to use	37 (5.6)	5 (0.7)	58 (9.6)	5 (0.8)	54 (6.1)	No variable	22 (3.3)
Mothers perceptions that this was the best brand ^1^	74 (11.3)	191 (28.6)	118 (19.5)	100 (16.7)	568 (64.3)	131 (21.8)	557 (82.6)
Mothers perception that this was closest to breastmilk ^2^	100 (15.3)	103 (15.4)	140 (23.2)	128 (21.3)	399 (45.1)	120 (19.9)	346 (51.3)
Other	8 (1.2)	0	2 (0.3)	12 (2.0)	27 (3.1)	52 (8.6)	0

^1^Mothers perception it is the best brand includes variables a) it is the best formula you can buy, b) it gives my baby the best start; c) it is good for baby’s health; d) it has special ingredients; and e) it is organic

^2^Mothers perception it is closest to breastmilk includes “it is the purest and most natural”

### Attitudes to infant feeding

In order to explore participants attitudes to CMF and breast feeding, participants were given a series of statements about formula feeding and requested to agree or disagree (Table 6). Statements included: ‘Breastmilk is best for babies’; ‘Formula keeps babies fuller for longer’; ‘Formula fed babies grow better than breastfed babies’. Most mothers in all countries agreed that breastmilk was the best choice for the baby, that breastfed babies were healthier and that breastfeeding helped the mother to bond with her baby. However, in all countries, except the UK, most mothers agreed that formula feeding was the best choice if the mother was planning to go back to work, ranging from 91.9% in Viet Nam to 71.0% in Morocco. In the UK only 30.3% of mothers agreed with this statement. However, a range of perceptions about formula feeding were widely held by participants in all countries, including that formula keeps babies fuller for longer, that formula feeding allows you to get your life back, formula helps babies to sleep better and many mothers expressed agreement that women should not feel pressured to breastfeed. A number of mothers in all countries agreed with statements that breastfeeding and formula feeding provide the same health benefits, and that CMF is similar to breastmilk (Table 6).

**Table 6:** Participants attitudes about infant feeding

	Bangla- desh ***N*=1178** **n (%)**	Mexico ***N*=1050** **n (%)**	Morocco ***N*=1050** **n (%)**	Nigeria ***N*=1050** **n (%)**	South Africa ***N*=1050** **n (%)**	United Kingdom ***N*=1050** **n (%)**	Viet Nam ***N*=1050** **n (%)**
***Proportion of participants who agreed with the following statements***							
Formula feeding is the better choice if a mother plans to go back to work	1002 (85.1)	892 (85.0)	746 (71.0)	836 (79.6)	799 (76.1)	318 (30.2)	965 (91.9)
Breastfeeding is best for your baby	1167 (99.1)	1009 (96.1)	857 (81.6)	960 (91.4)	832 (79.2)	787 (74.8)	930 (88.6)
Formula fed babies grow better than breastfed babies	421 (35.7)	186 (17.7)	441 (42.0)	291 (27.7)	391 (37.2)	48 (4.6)	423 (40.3)
Breastfeeding and FF provide a baby with the same health benefits	277 (23.5)	477 (45.4)	586 (55.8)	461 (43.9)	559 (53.2)	286 (27.2)	594 (56.6)
Formula helps babies sleep better	646 (54.8)	471 (44.9)	708 (67.4)	729 (69.4)	662 (63.0)	347 (33.0)	667 (63.5)
Formula is similar to breastmilk	148 (12.6)	336 (32.0)	461 (43.7)	494 (47.0)	513 (48.9)	298 (28.3)	417 (39.7)
Breastfeeding encourages better mother baby bonding	1141 (96.9)	996 (94.9)	875 (83.3)	975 (92.9)	839 (79.9)	775 (73.7)	1003 (95.5)
Formula keeps babies fuller for longer	829 (70.4)	731 (69.6)	739 (70.4)	795 (75.7)	716 (68.2)	551 (52.4)	755 (71.9)
Breastfeeding in public is embarrassing	856 (72.7)	198 (18.9)	677 (64.5)	191 (18.2)	292 (27.8)	177 (16.8)	311 (29.6)
Breastfed babies are healthier than formula fed babies	800 (67.9)	824 (78.5)	834 (79.4)	849 (80.9)	716 (68.2)	302 (28.7)	705 (67.0)
Formula feeding allows you to get your life back quicker	No variable	644 (61.3)	649 (61.8)	705 (67.1)	720 (68.6)	492 (46.8)	904 (86.1)
Breastfeeding is traditional / old fashioned	669 (56.8)	605 (57.6)	480 (45.7)	132 (12.6)	202 (19.2)	30 (2,9)	933 (88.9)
Breastfeeding helps you get your body shape back quicker	680 (67.7)	619 (59.0)	787 (75.0)	691 (65.8)	540 (51.4)	533 (50.7)	819 (78.0)
My partner prefers me not to breastfeed	54 (4.6)	156 (14.9)	379 (36.1)	223 (21.2)	303 (28.9)	51 (4.8)	83 (7.9)^1^
Formula feeding means I can leave my baby with others/ partner	932 (79.1)	601 (57.2)	643 (61.2)	796 (75.8)	864 (82.3)	780 (74.1)	926 (88.2)
There should be much more support to help women breastfeed successfully	1157 (98.2)	976 (93.0)	867 (82.6)	966 (92.0)	786 (74.9)	878 (83.5)	966 (92.0)
All brands of formula contain the same ingredients	No variable	111 (10.6)	No variable	No variable	No variable	No variable	No variable
You shouldn’t feel pressurized to breastfeed	507 (43.0)	681 (64.9)	838 (79.8)	850 (81.0)	727 (69.2)	1006 (95.6)	891 (84.9)

^1^In Viet Nam this question was phrased ‘men don’t like women breastfeeding’

## Discussion

This multi-country study, using a market research design, found widespread exposure of pregnant women and mothers to CMF marketing despite global recognition that marketing undermines breastfeeding practices [9, 18], and the provisions of the Code that aim to restrict CMF marketing practices and were endorsed by the World Health Assembly in 1981 [6]. Rates of exposure to CMF marketing activities varied across the seven countries, as did the type and location of CMF marketing, with more traditional settings such as television, magazines, supermarkets and shops being common sites of advertising. However, electronic marketing, particularly social media and YouTube, significantly contributed to marketing exposure in all settings. The findings are consistent with other reports that demonstrate the mechanisms by which CMF marketing seeks to change society’s wider perceptions of CMF, influencing the societal discourse, normalizing bottle feeding, and trying to create the sense of equivalence to breastmilk [1, 19]. The findings illustrate the enormous scope and reach and, therefore, the influence of marketing to promote CMF with the consequence of undermining breastfeeding practices globally [7, 9].

Unsolicited approaches from representatives of CMF companies were reported by mothers and pregnant women in all countries, most frequently in the UK and Viet Nam, where almost half of women reported receiving pop-up advertising on social media. Social media was the most common site of unsolicited contact by CMF companies and is becoming increasingly common [1]. Online communication greatly enhances ways for CMF companies to identify and approach women and families, and target them with personalized sympathetic communications designed to develop individual relationships and exploit their concerns [7, 20, 21]. Online baby clubs and mother support groups provide an opportunity for CMF companies using predatory marketing to insidiously influence the discourse [22], with the potential to use covert methods [1, 23]. The social media space is complex to regulate and monitoring violations of the Code is challenging [21, 24]. Social media platforms should also take accountability for content that violates the Code and may be harmful [22].

Health facilities were identified in five countries as locations where women frequently saw CMF marketing, and participants in all seven countries reported having received free samples of CMF either in or outside hospitals. Health professionals were reported to be the most common source of advice to formula feed by women in all country settings, except Viet Nam. Health professionals are highly respected sources of infant feeding advice [13], and this advice is likely to have a powerful effect on mothers’ infant feeding choices [25, 26]. The CMF industry understands the trusted role of all categories of health professionals, and marketing approaches therefore target them specifically because of the influence they have with families and within communities. Several approaches have been described in the literature as to how marketing engages health professionals: sponsoring medical meetings and conferences, funding research, providing free gifts or meals, funding education [1, 16, 27, 28]. Marketing to health professionals thereby creates conflicts of interest and may consciously or subconsciously influence the counselling they provide to mothers and families. Health authorities and professional bodies need to recognize these strategies for what they are and implement measures to protect rather than judge health professionals [25].

The findings further demonstrate that health professionals are the most trusted sources of information about infant feeding in all countries. This is aligned with other reports that illustrate how the CMF industry has sought to use and pathologize normal infant behavior such as sleep and crying to justify the use of CMF as a “solution” or to suggest equivalence of CMF with breastmilk [9, 13]. In this context, the creation of a range of specialist milks form the basis for marketing approaches that these products are required by some infants [8, 27, 28]. The marketing approaches create dilemmas for parents without medical expertise to make informed decisions and are mirrored by approaches to health professionals to influence their use of these products as solutions to common feeding problems described by parents [7, 12, 25]. Other products were marketed as having ‘added’ ingredients, usually ingredients that are obligatory, to suggest that CMF has particular benefits and is equivalent to breastmilk, when in fact all the ingredients are legislated and are essentially the same [1, 7]. These findings highlight the need for the medical and nursing professions, who are sometimes naïve to these strategies, to critically review their relationships with CMF companies and to comply with related Code legislation. In this context, health professional bodies should take measures to protects their members through training regarding conflicts of interest and marketing strategies [12, 27].

The National Implementation of the International Code status report of 2024 outlines the legal status of the Code in each country, including the extent to which provisions have been incorporated into national legislature. The report categorizes country’s legal measures as follows: 1) no legal measures included, 2) some provisions of the Code included, 3) legal measures moderately aligned with the Code, and 4) legal measures substantially aligned with the Code [29]. We reviewed the status of alignment with the Code across the seven countries in this study and found Bangladesh, Nigeria, South Africa and Viet Nam substantially aligned to the Code, Mexico was moderately aligned to the code, UK has some provisions of the Code included and Morocco has no legal measures included in the Code. However, countries being aligned with the Code did not preclude mothers experiencing exposure to CMF products. High levels of exposure to CMF marketing was found in both Viet Nam (substantially aligned) and UK (some provisions of the Code) and least exposure to CMF was found in Morocco, where there are no legal measures of the Code [29]. This reflects a range of implementation and methodological factors. If countries have substantial legislation but there is inadequate investment to monitor and implement Code adherence then it is unlikely that unethical marketing practices will cease.

## Why this study is different – strengths and limitations

The use of a consumer-marketing approach to explore how marketing aims to influence infant feeding decisions is a specific strength of this study. The sampling approach adopted is commonly used in market research and means that infant feeding practices reported cannot be considered representative of specific settings. Marketing research gathers information to understand the target market and inform effective strategies to promote their products. The cross-sectional nature of the study also means that no direct relationship can be drawn between marketing exposure and infant feeding practice. Yet, by using these methods, we can better understand how marketing impacts on infant feeding decision-making, and improve public health messaging strategies that might counter these approaches. This study was overseen by a team of international infant feeding and child health research experts to ensure the methodology was robust and interpretation consistent with the findings.

## Conclusion

This study provides important quantitative data about womens’ exposure to CMF marketing in seven countries and insights into the techniques and strategies employed by CMF companies. CMF marketing has persuaded many families to use an ultra-processed product that is inferior to breastmilk and in many circumstances is harmful to children. Deliberate action is required across the political and health domains and by civil society if public health is to counter the sophisticated and highly resourced actions of CMF companies. While this includes increased enforcement and monitoring of the Code and actions to eliminate interactions between health practitioners and CMF companies, more is also needed to restore belief and confidence in the exceptional properties and benefits of breastfeeding. We suggest there is much to learn from the targeted and pragmatic techniques used in marketing research, particularly in social media, and that when public health designs approaches to foster changes toward healthy behavior, commercial marketers might be relevant partners in health.

## Supplementary Information

Supplementary Material 1.

Supplementary Material 2.

Supplementary Material 3.

Supplementary Material 4.

## Data Availability

No datasets were generated or analysed during the current study.

## Abbreviations

CAPI: computer-assisted personal interview
CMF: commercial milk formula
SES: socioeconomic status
UK: United Kingdom
WHO: World Health Organization
