# Supplementary material for: Women’s exposure to commercial milk formula marketing: a WHO multi-country market research study
Source: Global Health. 2024 Nov 28;20:85. doi: 10.1186/s12992-024-01088-y (PMC11603767; doi:10.1186/s12992-024-01088-y)
Supplement: Supplementary file 1 — Supplementary Material 1. [file 12992_2024_1088_MOESM1_ESM.docx]

APPENDIX 1

**Supplementary Table 1: Research locations in each country**

|  | **City 1** | **City 2** |
| --- | --- | --- |
| Bangladesh | Dhaka | Chittagong |
| Mexico | Mexico City | Guadalajara |
| Morocco | Marrakech | Rabat |
| Nigeria | Lagos | Abuja |
| South Africa | Johannesburg | Cape Town |
| United Kingdom | London | Glasgow |
| Viet Nam | Ho Chi Minh City | Hanoi |
