# Supplementary material for: Women’s exposure to commercial milk formula marketing: a WHO multi-country market research study
Source: Global Health. 2024 Nov 28;20:85. doi: 10.1186/s12992-024-01088-y (PMC11603767; doi:10.1186/s12992-024-01088-y)
Supplement: Supplementary file 2 — Supplementary Material 2. [file 12992_2024_1088_MOESM2_ESM.docx]

**APPENDIX 2**

**Supplementary Table 2: Sample size by population, feeding practice, socioeconomic group and location**

| Category | Age of baby | Socio economic group | **City 1** | **City 2** |
| --- | --- | --- | --- | --- |
| Pregnant | Pregnant | Low | 50 | 50 |
|  |  | Medium | 50 | 50 |
|  |  | High | 50 | 50 |
| Breastfeeding | 0-12 months | Low | 25 | 25 |
|  |  | Medium | 25 | 25 |
|  |  | High | 25 | 25 |
| Women who feed their children with commercial formula products | 0-3 months | Low | 25 | 25 |
|  |  | Medium | 25 | 25 |
|  |  | High | 25 | 25 |
|  | 4-6 months | Low | 25 | 25 |
|  |  | Medium | 25 | 25 |
|  |  | High | 25 | 25 |
|  | 7-12 months | Low | 25 | 25 |
|  |  | Medium | 25 | 25 |
|  |  | High | 25 | 25 |
|  | 13-18 months | Low | 25 | 25 |
|  |  | Medium | 25 | 25 |
|  |  | High | 25 | 25 |
