# Supplementary material for: Women’s exposure to commercial milk formula marketing: a WHO multi-country market research study
Source: Global Health. 2024 Nov 28;20:85. doi: 10.1186/s12992-024-01088-y (PMC11603767; doi:10.1186/s12992-024-01088-y)
Supplement: Supplementary file 3 — Supplementary Material 3. [file 12992_2024_1088_MOESM3_ESM.docx]

#

**PRE AND POSTNATAL WOMEN SURVEY**

**VIETNAM**


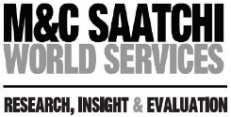


**NOVEMBER 2019**

**PRE AND POSTNATAL WOMEN SURVEY**

**INSTRUCTIONS TO INTERVIEWER**

**Items to carry with you during field work**

Each day before leaving for the field, all researchers should check that they have adequate supplies for the day and have carried out necessary procedures. This includes:

- A fully charged CAPI device, along with a briefcase or bag to carry it in
- Check the script is working
- The Interviewer and Supervisor Manual
- Identification documents (including a name badge)
- Interviewer call log
- Informed consent forms (in all necessary languages)
- Any personal items you will need to be comfortable
- SUPERVISORS: Interview observation sheet
- SUPERVISORS: Supervisor log sheet
- SUPERVISORS: Back-check sheet

**Pre-interview procedures**

- Introduce yourself, explain the research and its objectives, and inform the respondent of the contractor’s name and the purpose of the research
- Make sure participants are comfortable:
  - The questionnaire will be administered face-to-face, either at a clinic, the respondent’s home or another location by mutual agreement. Any alternative locations must be safe and quiet with minimal distractions, and no onlookers. If you are unsure about the safety or suitability of the location, please speak with your supervisor. If he/ she is unavailable, do not complete the interview.
  - Participants must feel safe and comfortable throughout the entire duration of the interview. Interviewers should be approachable and respectful, and women should never feel forced into taking part in the survey, or that their answers are being judged. Participants have the right to withdraw from the survey at any time.
- Please ensure that all participants have the opportunity to read the participant information sheet before they begin, and can ask questions.
- Please give instructions to the woman on what is meant by breastfeeding and formula feeding.
- Please inform the woman about how the data collected will be stored and used.

**Conducting an interview**

- Follow the interviewer instructions provided in the survey carefully. The words in bold are instructions for the enumerator and should not be read out to the respondents
- If the respondent needs to pause or cancel the interview, please find an alternative date and time to call back
- The questionnaire should be administered word for word, reading the whole question. If the respondent does not understand, repeat the whole question
  - Do not try to explain questions in your own words. If participants are still unsure of a question’s meaning, record their response as ‘don’t know’.
  - Do not give leading prompts (a statement/ explanation that suggests what the ‘right’ answer is)
- Record an answer for every question
  - If a respondent does not know the answer, or refuses to answer, this must still be recorded as it is important
    - NOTE: Do not read the option ‘don’t know’ or ‘refused’ to participants
  - If you do not understand a respondent’s answer, ask them to repeat or explain – do not guess or write down incorrect information
- Write down verbatim answers as spoken for all open-ended questions
  - Do not summarise answers or paraphrase
- Be patient with respondents - do not hurry the interview
  - If participants begin to speak or provide information about topics that are irrelevant to the study, it may be necessary to steer them back to the focus of the research – however, this must be done politely and respectfully

**Concluding the survey**

At the end of the survey, participants must be thanked for their time, they must be read the verbal consent script and given contact details of the field agency and contractor. You must record confirmation of verbal consent at the end of the survey.

**TO BE COMPLETED BY THE INTERVIEWER**

Interviewer name:

Field supervisor name:

Location of interview (house/ clinic):

Area of interview (town and city):

Date of interview (dd/mm/yyyy):

Time of interview (hh/mm am/pm):

**WOMAN’S U&A SURVEY SCREENER: MASTER**

**If consent is given, record the ID, respondent name, gender, age and contact details. Also record that you have collected the participants signature. Then continue with screening interview. If consent is not given, thank them for their time and interest.**

**Please record– YES if interviewed**

**NO if refused or does not qualify**

**INTERVIEWER - READ THE FOLLOWING**

*Hello, my name is* ***[INSERT INTERVIEWER NAME]****. Thank you for taking the time to speak with me today. I’d like to open our conversation with some questions about you*.

**CODE INTERVIEWER NUMBER, RESPONDENT NUMBER AND LOCATION OF INTERVIEW**

**Full Name ----------------------------------------**

**Email ----------------------------------------**

**Address ----------------------------------------**

**----------------------------------------**

**Phone ----------------------------------------**

**SAMPLING FRAME TO COME FROM ACORN**

**ASK ALL**

1. **Do you or does any member of your family or close friends work for any of the following companies?** **READ OUT OPTIONS, MULTICODE**

1. A market research company/ marketing company **CLOSE**

2. A company that makes infant formula milk **CLOSE**

3. A bank

4. An advertising agency **CLOSE**

5. A company that works with infant formula milk companies **CLOSE**

6. None of these

7. Don’t know **CLOSE**

**2. How old were you at your last birthday? SINGLE CODE**

1. Under 18 **CLOSE**
2. 18-24
3. 25-29
4. 30-34
5. 35-39
6. 40-44
7. 45-49
8. Over 50

Write in actual age ___________years

**ASK ALL**

**3. Can you speak, read, write and understand Vietnamese fluently? SINGLE CODE**

1. Yes
2. No **CLOSE**

**ASK ALL**

**4. Which of the following apply to you? MULTICODE**

1. I am pregnant (more than 3 months)
2. I have a baby aged 0-18 months
3. I don’t have a baby aged 0-18 months **CLOSE**
4. I am not (more than 3 months) pregnant **CLOSE**

**THANK AND CLOSE INTERVIEW IF CODES 3 AND 4**

**Interviewer please read out: We are now going to ask you some questions about infant feeding, we will use the following definitions:**

***Breastfeeding*** *refers to giving your baby breastmilk via the breast, bottle or cup*

***Formula feeding*** *refers to giving your baby formula in a bottle or cup*

**ASK IF CODES 1 AT Q3**

**5. How do you plan to feed your baby in the first two weeks? SINGLE CODE**

1. Breastfeeding only
2. Formula feeding only
3. Both breastfeeding and formula feeding from birth
4. Breastfeeding first and then switch to formula feeding
5. Breastfeeding first and then breastfeeding and giving formula
6. Breastfeeding and formula feeding first and then switch to formula feeding
7. I haven’t planned yet/ don’t know **CLOSE INTERVIEW**

**ASK IF CODES 2 AT Q3, OTHERS SKIP TO Q7**

**6.How old is your youngest baby (that is 0 -18 months)? SINGLE CODE**

1. One month or younger

2. 2 - 3 months

3. 4 - 6 months

4. 7 - 9 months

5. 10 -12 months

6. 13 -18 months

**7. How are you currently feeding your youngest baby? SINGLE CODE**

- 1. Breastfeeding only since birth
  2. Formula feeding only since birth
  3. Both breastfeeding and formula feeding from birth
  4. Breastfed first and now I am formula feeding
  5. Breastfed first and now I am breastfeeding and giving formula
  6. Breastfeeding and formula feeding first, and now giving formula

**IF CODES Q5 6 AND Q6 1 AND NOT PREGNANT, CLOSE INTERVIEW. BREASTFEEDING ONLY WOMEN MUST HAVE A BABY AGED 0-12 MONTHS**

**ASK IF CODES 2,3,4,5 OR 6 AT Q6 (MOTHERS) OR 2,3,4,5 OR 6 AT Q4 (PREGNANT)**

**8. Do you or your baby have any health or other issues that prevents, or would prevent you from breastfeeding?**

1. Yes **THANK & CLOSE**
2. No
3. Don’t know

**ASK IF CODES 4,5 OR 6 AT Q6**

**9. How old was your youngest baby when you started formula feeding?**

**SINGLE CODE**

1. A day or less
2. 2-6 days
3. 1-2 weeks
4. 3-4 weeks
5. 1-2 months
6. 3-4 months
7. 5-6 months
8. 7-9 months
9. 10-12 months
10. More than 12 months

**ASK ALL WHO ARE PREGNANT (Screener Q3 code 1)**

**10. How many weeks pregnant are you?**

1. 0-13 weeks (first trimester) **ONLY RECRUIT IF HAS BABY 0-18MTHS (Q3/2)**
2. I4-26 weeks (second trimester)
3. 27-42 weeks (third trimester)
4. I am not sure **ONLY RECRUIT IF HAS BABY 0-18MTHS (Q3/2)**

**ASK ALL**

**11. I am now going to read you a list of things that you or your household may have. For each, please tell me if you have one. SINGLE CODE FOR EACH**

|  | **1**  **Yes**  **(present)** | **2**  **No**  **(not present)** | **3**  **Don’t know** | **4**  **Prefer not to say** |
| --- | --- | --- | --- | --- |
| 1. Laptop computer | 1 | 2 | 3 | 4 |
| 2. Bank account | 1 | 2 | 3 | 4 |
| 3. Mattress | 1 | 2 | 3 | 4 |
| 4. Refrigerator | 1 | 2 | 3 | 4 |
| 5. Television | 1 | 2 | 3 | 4 |
| 6. Motorbike | 1 | 2 | 3 | 4 |
| 7. Mobile with email facility/ smartphone | 1 | 2 | 3 | 4 |
| 8. Air conditioning (built in NOT portable) | 1 | 2 | 3 | 4 |
| 9. Dining table | 1 | 2 | 3 | 4 |
| 10. Motorcar | 1 | 2 | 3 | 4 |

**ASK ALL**

**12. What is the income category that best describes the monthly income of your household (please include all income sources and this should be the amount that you receive after taxes etc.)? SINGLE CODE**

1. 0 - 4,000,000 VND **LOW SES**
2. 4,000,001 – 9,000,000 VND **LOW SES**
3. 9,000,001 – 14,000,000 VND **MID SES**
4. 14,000,001 – 20,000,000 VND **MID SES**
5. 20,000,000 VND + **HIGH SES**

**REFER TO QUOTAS – HIGH SES – CODE 5, MEDIUM SES – CODE 3 OR 4, LOW SES – 1 OR 2**

**13. How many other (than your youngest baby under 18 months) living children (MOTHERS)/ living children (PREGNANT), if any, do you have? SINGLE CODE**

1. None
2. One
3. Two
4. Three
5. Four
6. Five
7. Six
8. Seven
9. Eight
10. Nine
11. Ten or more

**14. What is your marital status? SINGLE CODE**

1. Single
2. Married
3. Cohabiting
4. Divorced/separated
5. Widowed

**15. Which of these ethnic groups best describes you? SINGLE CODE**

1. Vietnamese
2. Chinese origin
3. Others

**16. Which of the following have you done in the past six months? You can say as many as apply. MULTI CODE**

1. Been to a movie
2. Been to a job interview
3. Eaten at a restaurant
4. Attended a group discussion, or been interviewed for research related to mothers and babies **CLOSE**
5. Been to a wedding
6. None of these

**IF RESPONDENT FULFILS A REQUIRED QUOTA, ALLOCATE INTERVIEW TO TABLE BELOW AND CONTINUE WITH FULL SURVEY, OR ARRANGE A FUTURE DATE AND TIME FOR COMPLETION AND RECORD BELOW. OTHERWISE THANK AND CLOSE INTERVIEW**

| **TYPE OF WOMEN** | **AGE OF BABY** | **SES**  **(Q10/11)** | **CODE** |
| --- | --- | --- | --- |
| Pregnant  **Q3/1** | NA | Low | 1 |
|  |  | Medium | 2 |
|  |  | High | 3 |
| Breastfeeding women (exclusive)  **Q3/2 and Q6/1** | 0-12 months  **Q5/1,2,3,4 or5** | Low | 4 |
|  |  | Medium | 5 |
|  |  | High | 6 |
| Women who feed their children BMS products (exclusive or mixed)  **Q3/2**  **Q6/2,3,4,5 or 6** | 0-3 months  **Q5/1 or 2** | Low | 7 |
|  |  | Medium | 8 |
|  |  | High | 9 |
|  | 4-6 months  **Q5/3** | Low | 10 |
|  |  | Medium | 11 |
|  |  | High | 12 |
|  | 7-12 months  **Q5/4 or 5** | Low | 13 |
|  |  | Medium | 14 |
|  |  | High | 15 |
|  | 13- 18 months  **Q5/6** | Low | 16 |
|  |  | Medium | 17 |
|  |  | High | 18 |

Interview date___________________Time _______**CAPI WILL RECORD LENGTH OF INTERVIEW**

Interview arranged for future date Date___________________Time____________

**WOMAN’S U&A MAIN SURVEY: VIETNAM**

**INTERVIEWER - READ THE FOLLOWING**

*Hello, my name is* ***[INSERT INTERVIEWER NAME]****. Thank you for taking the time to speak with me today. I’d like to open our conversation with a few more questions about yourself and your household*.

**ASK ALL Q1-6**

**1. How many adults live in your household in total?**

Write in number ___________

**2. And how many children under 18 years old live in your household?**

Write in number ___________

**SCRIPTER CREATE A TOTAL VARIABLE BY ADDING VALUE AT 1 + 2**

**4. What is the highest level of education that you have completed?**

**e.g school/ college/ university? SINGLE CODE**

1. Primary (up to the age of 11)
2. Secondary (up to the age of 16)
3. Secondary (up to the age of 18)
4. Higher education – university or college (over age of 18)
5. Other
6. No education

**5. How many bedrooms are there in your household?**

Write in number _________________________

**6. Are you currently working? SINGLE CODE**

1. I work full time (30 hours + per week)
2. I work part time (8-29 hours per week)
3. I work part time (less than 8 hours per week)
4. I am a housewife at home/ full time mother
5. I am on maternity leave
6. I am unemployed / looking for a job
7. I am in full time education

**ASK IF 1,2,3,5 AND 7 IN Q6**

**7. Are you receiving any paid maternity support?**

1. Yes
2. No

**ASK IF HAS A BABY OF 0-18 MONTHS Screener Q4 CODE 2 AND IS NOT WORKING/ MATERNITY LEAVE Q6 CODE 4,5,6 OR 7,8 OTHERS GO TO Q12**

**8. Are you planning to work within the next year?**

1. Yes
2. No
3. Not sure

**ASK ALL**

**9.Which district do you live in?**

| **HCMC** | **Hanoi** |
| --- | --- |
| Quận 1 | Ba Đình |
| Quận 12 | Hoàn Kiếm |
| Quận Thủ Đức | Tây Hồ |
| Quận 9 | Long Biên |
| Quận Gò Vấp | Cầu Giấy |
| Quận Bình Thạnh | Đống Đa |
| Quận Tân Bình | Hai Bà Trưng |
| Quận Tân Phú | Hoàng Mai |
| Quận Phú Nhuận | Thanh Xuân |
| Quận 2 | Bắc Từ Liêm |
| Quận 3 | Nam Từ Liêm |
| Quận 10 | Hà Đông |
| Quận 11 | Sóc Sơn |
| Quận 4 | Đông Anh |
| Quận 5 | Gia Lâm |
| Quận 6 | Thanh Trì |
| Quận 8 | Mê Linh |
| Quận Bình Tân | Sơn Tây (township) |
| Quận 7 | Ba Vì |
| Huyện Củ Chi | Phúc Thọ |
| Huyện Hóc Môn | Đan Phượng |
| Huyện Bình Chánh | Hoài Đức |
| Huyện Nhà Bè | Quốc Oai |
| Huyện Cần Giờ | Thạch Thất |
|  | Chương Mỹ |
|  | Thanh Oai |
|  | Thường Tín |
|  | Phú Xuyên |
|  | Ứng Hòa |
|  | Mỹ Đức |
|  |  |

**10a Did you give birth (MOTHERS Screener Q4 CODE 2)/ Do you plan to give birth (PREGNANT Screener Q4 CODE 1) in a hospital?**

1. Yes, at public hospital
2. Yes, at private hospital
3. No

**ASK IF YES**

**10b. Which hospital did you give birth in (MOTHERS Screener Q4 CODE 2)/ do you plan to give birth in? (PREGNANT Screener Q4 CODE 1)**

**WE HAVE A LIST OF HOSPITALS FOR CODING IN VIETNAM**

**ASK ALL**

**11. Was your youngest baby delivered by caesarean section (MOTHERS Screener Q4 CODE 2)**/**Do you plan to have your baby by caesarean section (PREGNANT Screener Q4 CODE1)?**

1. Yes
2. No

________________________________Write in

**____________________________________________________________________________**

**FEEDING YOUR BABY**

**Interviewer read out to MOTHERS (Screener Q4 CODE 2):**

**Thank you for telling us a bit about yourself, we would now like to ask you some questions about how you feed your youngest baby (under 18 months)**

**IF PREGNANT (Screener Q4 CODE 1) SKIP TO Q19**

**ASK IF CODES 4 or 6 AT Screener Q7, OTHERS SKIP TO Q14**

**12. How long did you breastfeed your baby for, before you switched to formula feeding? SINGLE CODE**

1. A day or less
2. 2-6 days
3. 1-2 weeks
4. 3-4 weeks
5. 1-2 months
6. 3-4 months
7. 5-6 months
8. 7-9 months
9. 10-12 months
10. More than 12 months

**ASK IF CODES 4 AT Screener Q7**

**13. Which of the following applies to you? SINGLE CODE**

1. I introduced formula before I stopped breastfeeding
2. I moved from breastfeeding only to feeding formula only

**You mentioned earlier in the survey that you ………** **(SCRIPTER INSERT ANSWER AT SCREENER Q7 – HOW FEEDING)**

**14. Is this how you originally planned to feed your baby? SINGLE CODE**

- 1. Yes
  2. No
  3. I didn’t have a set plan for feeding my baby

**If q14 =1 (yes) then skip to Q17**

**If q14=2 (no) then ask Q15 and then skip Q17**

**ASK IF ANSWERS ‘NO’ CODE 2 at Q14**

**15. Overall, how does this differ from your original plan for feeding your baby DO NOT PROMPT?** **CODE ALL THAT APPLY**

1. I have breastfed more/ for longer than I expected
2. I have fed formula more/ for longer than I expected
3. I have not breastfed at all
4. I have not fed formula at all
5. Other (please write in answer)

**ASK ALL MOTHERS (Screener Q4 CODE 2)**

**16. Are you expecting to continue feeding your baby the same type of milk over the next month? SINGLE CODE**

1. Yes
2. No, I intend to use a Stage 1 formula and breast milk
3. No, I intend to use Stage 2/3/4 formula and breast milk
4. No, I intend to use another formula and breast milk
5. No, I intend to use Stage 1 formula only
6. No, I intend to use Stage 2/3/4 formula only
7. No, I intend to use another formula only
8. No, I intend to stop feeding formula to my baby
9. No, I intend to feed my baby cows, goats or other milks
10. No, I intend to stop feeding milk to my baby
11. I haven’t decided yet
12. Other (please write in)____________________________________

**ASK IF CODED 2 AT Screener Q7 (FORMULA FROM BIRTH) SINGLE CODE**

**17.Have you ever breastfed your youngest baby?**

1. Yes
2. No

**ASK IF CODED Screener Q13 NOT 1, OTHERS SKIP TO Q19**

**18. How did you feed your previous youngest child (MOTHERS) youngest child (PREGNANT)?**

1. Breastmilk only
2. Formula milk only
3. Both breastmilk and formula milk from birth
4. Breastmilk first and then switched to formula milk
5. I breastfed first and then I breastfed and gave formula
6. I Breastfed and formula fed first and then switched to formula feeding only

**INFORMATION ABOUT INFANT FEEDING**

**Interviewer read out to all: I would now like to ask about the sources of information that you use and have used, for advice on pregnancy, birth, mothering and baby care. Then we will talk a bit more about your own experiences and views on feeding your baby.**

**ASK ALL**

**19. Please tell me which of the following you have ever used/use for information specifically about feeding your baby or infant?** **MULTICODE, ROTATE ORDER OF LIST, BUT KEEP BLOCKS OF ANSWERS TOGETHER**

**NOTE TO INTERVIEWER:** DO NOT SHOW TABLET, LET RESP ANSWER SIMULTANEOUSLY AND INTERVIEW TO CHOOSE CODES.

**SCREEN SHOULD NOW JUST SHOW THOSE MENTIONED AT Q19**

**20. And which are the top three most useful sources of information about feeding your baby or infant that you have used/use, please rank top three? SINGLE CODE FOR EACH**

| **SOURCE** | **Q19** | **Q20 1ST** | **Q20 2ND** | **Q20 3RD** |
| --- | --- | --- | --- | --- |
| Books | 1 | 1 | 1 | 1 |
| Magazines | 2 | 2 | 2 | 2 |
| Youtube | 3 | 3 | 3 | 3 |
| Newspapers | 4 | 4 | 4 | 4 |
| Leaflets and printed information handed out at visits to health centres/clinics | 5 | 5 | 5 | 5 |
| Radio | 6 | 6 | 6 | 6 |
| TV | 7 | 7 | 7 | 7 |
| Friends | 8 | 8 | 8 | 8 |
| Family members | 9 | 9 | 9 | 9 |
| Other mothers | 10 | 10 | 10 | 10 |
| Phone help lines | 11 | 11 | 11 | 11 |
| In-person parent support groups | 12 | 12 | 12 | 12 |
| Health professional | 13 | 13 | 13 | 13 |
| Religious Leaders/Traditional Leaders | 14 | 14 | 14 | 14 |
| App on my phone | 15 | 15 | 15 | 15 |
| Social media (Instagram, Facebook, Twitter, other) | 16 | 16 | 16 | 16 |
| Google | 17 | 17 | 17 | 17 |
| Producer websites | 18 | 18 | 18 | 18 |
| Distributor/store websites | 19 | 19 | 19 | 19 |
| Medical websites | 20 | 20 | 20 | 20 |
| Other websites | 21 | 21 | 21 | 21 |
| Other (please specify) **KEEP AT END OF LIST** | 22 | 22 | 22 | 22 |
| None of the above **KEEP AT END OF LIST** | 23 | 23 | 23 | 23 |

**FACTORS INFLUENCING FEEDING**

**Interviewer read out to all: We are now going to ask you a bit more about the important factors that may have influenced how you feed your baby.**

**ASK ALL**

**21. Interviewer read out: Have any of the following individuals or groups given advice on, or recommended formula feeding or specific brands of formula milk to you? SHOW LIST ON SCREEN, MULTICODE AS MANY AS APPLY**

|  | **Q21** |
| --- | --- |
| 1. My spouse/partner/ husband | 1 |
| 1. My mother/ mother in law | 2 |
| 1. Other people in my family | 3 |
| 1. My close friends | 4 |
| 1. People I talk to on social media/chat forums | 5 |
| 1. People in my community | 6 |
| 1. People I watch or follow on TV/Radio/social media | 7 |
| 1. Scientific experts | 8 |
| 1. Salesperson | 9 |
| 1. Doctor/Consultant/Paediatrician | 10 |
| 1. Midwife/Nurse | 11 |
| 1. Pharmacist | 12 |
| 1. Community leaders e.g. Religious or traditional leaders | 13 |
| 1. Prenatal class | 14 |
| 1. Other (specify) | 15 |

**ASK ALL**

**22. Interviewer read out: Which of the following factors, if any, were important to you when deciding how to feed your baby (MOTHERS Screener Q4/2) /were important to you when planning how to feed your baby (PREGNANT Screener Q4/1)? You can choose as many as apply MULTICODE SHOW RESPONDENT LIST ON SCREEN**

**SHOW ONLY THOSE CONSIDERED IMPORTANT AT 22**

**23. And of those factors which were at all important, please rank the top three most important factors that contributed to your decision on how you feed your baby/ or how you plan to feed your baby. IF ONLY 2 MENTIONED, RANK THEM, IF ONLY ONE, PUT AS TOP, ALLOW RESPONDENT TO VIEW LIST ON SCREEN**

|  | **Q22 Important** | **Q23 1^ST^ most important** | **Q23 2ND**  **most important** | **Q23 3RD most important** |
| --- | --- | --- | --- | --- |
| 1. It is convenient | 1 | 1 | 1 | 1 |
| 1. It allows me to do my work | 3 | 3 | 3 | 3 |
| 1. It is beneficial for the health and development of my child | 4 | 4 | 4 | 4 |
| 1. It gives me health benefits | 5 | 5 | 5 | 5 |
| 1. It is an affordable | 7 | 7 | 7 | 7 |
| 1. I have seen advertising that suggests this is a good way to feed | 8 | 8 | 8 | 8 |
| 1. It will help me to establish a routine | 9 | 9 | 9 | 9 |
| 1. It will help me to get my body back in shape | 10 | 10 | 10 | 10 |
| 1. It’s the way that most people feed their baby | 11 | 11 | 11 | 11 |
| 1. My friends and family approve | 12 | 12 | 12 | 12 |
| 1. It’s the way that websites and forums say that you should feed a baby | 13 | 13 | 13 | 13 |
| 1. It is the best way to feed your baby | 15 | 15 | 15 | 15 |
| 1. A health professional suggested | 16 | 16 | 16 | 16 |

**ASK ALL**

**24. How do the majority of your close friends and family feed their baby in the first months of their life?**

1. Most breastfeed only
2. Most use formula milk only
3. Most breastfeed and use formula from birth
4. Most breastfeed first and then switch to formula feeding
5. Most breastfeed first and then breastfeed and give formula
6. Most breastfeed and give formula first and then switch to using formula
7. I don’t know

**KNOWLEDGE AND USE OF FORMULA BRANDS**

**Interviewer read out: We are now going to ask you about the varieties of formula and also the specific brands and sub brands that you know.**

**ASK ALL WHO HAVE FED FORMULA TO A BABY**

**ASK IF SCREENER (Q7/ 2,3,4,5 or 6) OR (SCREENER Q13 NOT 1 AND Q18/2,3,4,5 or 6)**

**SHOW BRANDS. SINGLE CODE BELOW**

**25 Which brand(s) of formula milk have you ever used? MULTICODE**

**ASK IF PREGNANT WITH FIRST BABY, PLANNING TO FEED FORMULA**

**(SCREENER Q4/1 AND Q13/1 and Q5/2,3,4,5 or 6)**

**26 Which brand(s) of formula milk are you planning to use? MULTICODE**

**ASK ALL, SHOW ALL BRANDS AND IMAGES**

**27 Thinking about all the brands of formula milk that you have heard of, please can you say which brand of formula you think is best, it doesn’t matter whether you have used this brand or not? SINGLE CODE BELOW**

|  | **EVER USE Q25** | **WOULD USE Q26** | **BEST Q27** |
| --- | --- | --- | --- |
| Alpha Lipid Lifeline | 1 | 1 | 1 |
| Aptamil | 2 | 2 | 2 |
| Dielac | 3 | 3 | 3 |
| Dumex | 4 | 4 | 4 |
| Dutch lady/Bella Holandesa | 5 | 5 | 5 |
| Enfagrow | 6 | 6 | 6 |
| Enfamil | 7 | 7 | 7 |
| Ensure | 8 | 8 | 8 |
| Friso | 9 | 9 | 9 |
| Grow | 10 | 10 | 10 |
| Lactogen | 11 | 11 | 11 |
| Meiji | 12 | 12 | 12 |
| Nan | 13 | 13 | 13 |
| Nutifood | 14 | 14 | 14 |
| Pediasure | 15 | 15 | 15 |
| Similac | 16 | 16 | 16 |
| ColosMama | 17 | 17 | 17 |
| Blackmores | 18 | 18 | 18 |
| Other (specify)  __________________  __________________ | 19 | 19 | 19 |
| Don’t know/unsure | 20 | 20 | 20 |

**ASK ALL**

**28. Why do you think that this brand is the best? DO NOT PROMPT, PROBE ‘ANYTHING ELSE’**

Write in _____________________________________________________________________________

**29. How did you first hear about ____________? SINGLE CODE FOR EACH**

1. I was given this brand in hospital/health clinic

2. It was recommended to me by a health professional

3. I was given a free sample outside of hospital/health clinic

4. I received a promotion

5. It was the one I could afford

6. My friends and/ or family use this brand

7. I had used this brand before

8. It is the best formula you can buy

9. It’s good for the baby’s health

10.It is the closest formula to breast milk

11.It has special ingredients

12.I saw an advert for it e.g. in a magazine or on the television

13.It was discussed on social media

14.It was the only one available

15.It is the most suitable for my baby’s condition

16.I haven’t decided which formula to use

17.Other ______________ Write in

**ASK ALL WHO HAVE FED FORMULA TO A BABY OR ARE PLANNING TO**

**SCREENER Q7/ 2,3,4,5 or 6 OR (SCREENER Q5/ 2,3,4,5 OR 6 AND NOT Q26/20) OR Q12/NOT 1 AND Q18/2,3,4,5 OR 6**

**30. We would like to understand how you made the decision as to which brand of formula to give your baby. Please read through these options and select as many as apply to you. MULTICODE**

1. I was given this brand in hospital/health clinic
2. It was recommended to me by a health professional
3. I was given a free sample outside of hospital/health clinic
4. I received a promotion
5. It was the one I could afford
6. My friends and/ or family use this brand
7. I had used this brand before
8. It is the best formula you can buy
9. It’s good for the baby’s health
10. It is the closest formula to breast milk
11. It has special ingredients
12. I saw an advert for it e.g. in a magazine or on the television
13. It was discussed on social media
14. It was the only one available
15. It is the most suitable for my baby’s condition
16. I haven’t decided which formula to use
17. Other ______________ Write in

**ASK ALL**

**31. What do you think are the benefits, if any, of using formula milk?** **DO NOT PROMPT, WRITE ANSWERS IN FULL**

1. ………………………...Open ended
2. None
3. Don’t know

**ASK ALL, DO NOT PROMPT**

**32. Have you heard of Stage 2 formula?**

1. Yes

2. No

3. Don’t know

**33. What age of baby** **do you think Stage 2 formula is aimed at? MULTICODE, INTERVIEWER CODE INTO RELEVANT SCALE E.G IF SAYS 7-12 MONTHS THEN CODE 3 AND 4**

1. 0-3 months
2. 4-6 months
3. 7-9 months
4. 10-12 months
5. 13-18 months
6. Older than 18 months

**SHOW STAGE 2 COLLAGE STIMULUS**

**34.** **Do you think that a baby needs Stage 2 formula? SINGLE CODE**

1. Yes
2. No
3. Don’t know

**ASK IF 34 CODE 1**

**35. How did you find out that a baby needs Stage 2 formula?**

1. I was told so by a friend
2. I was told so by another mother
3. I was told so by a family member
4. I was told so by a medical professional
5. I read information on it
6. I saw an advert showing the benefits
7. Other (please specify…)
8. Don’t know

**ASK ALL, DO NOT PROMPT**

**36. What do you think are the reasons for using Stage 2 formula? CODE INTO RESPONSES BELOW AND WRITE IN OTHERS. MULTICODE**

1. Hunger
2. Stops baby crying
3. For iron
4. It’s good for sleep
5. It benefits brain development
6. It’s good for the baby’s future
7. It helps babies grow
8. Cow’s milk doesn’t have enough nutrients
9. Some infants are allergic to dairy
10. Breast milk cannot provide enough nutrients in this stage
11. Other **PLEASE WRITE IN _____________________________________________**
12. I don’t know any reason/ no reason

**37. Have you heard of Stage 3 or 4 formula?**

1. Yes

2. No

3. Don’t know

**ASK ALL, DO NOT PROMPT**

**38. What age of baby** **do you think Stage 3 or 4 formula is aimed at? MULTICODE, INTERVIEWER CODE INTO RELEVANT SCALE E.G IF SAYS 7-12 MONTHS THEN CODE 3 AND 4**

1. 0-3 months
2. 4-6 months
3. 7-9 months
4. 10-12 months
5. 13-18 months
6. Older than 18 months

**ASK ALL, SHOW STAGE 3/4 COLLAGE STIMULUS**

**39. Do you think that a baby or child needs Stage 3 or 4 formula?**

1. Yes

2. No

3. Don’t know

**ASK IF CODES YES AT Q39, OTHERS GO TO 41**

**40. How did you find out that a baby needs Stage 3 or 4 formula?**

1. I was told so by a friend
2. I was told so by another mother
3. I was told so by a family member
4. I was told so by a medical professional
5. I read information on it
6. I saw an advert showing the benefits
7. Other (please specify…)
8. Don’t know

**ASK ALL DO NOT PROMPT**

**41. We would now like you to tell us in your own words, what do you think are the reasons why somebody would feed Stage 3 or 4 formula to their child? MULTICODE CODE INTO RESPONSES BELOW OR WRITE IN**

1. For iron
2. Hunger
3. Stops baby crying
4. It’s good for sleep
5. It benefits brain development
6. It helps babies grow
7. It’s good for the baby’s future
8. Cow’s milk doesn’t have enough nutrients
9. Some infants are allergic to dairy
10. Breast milk cannot provide enough nutrients in this stage
11. Other **PLEASE WRITE IN** _____________________________________________
12. I don’t know any reason/ no reason

**ASK ALL**

**42. Which of these varieties of formula have you ever fed/ or intend to feed to your baby or child? MULTI CODE**

|  | **Q42 EVER FED/ PLAN TO FEED** |
| --- | --- |
| Stage 1- Infant formula | 1 |
| Stage 2 - Follow on formula | 2 |
| Stage 3 or 4 - Toddler formula | 3 |
| Tummy troubles formula | 4 |
| Anti-reflux formula | 5 |
| Lactose free formula | 6 |
| Allergy formula | 7 |
| Good night formula | 8 |
| Hungry baby formula | 9 |
| Organic formula | 10 |
| Other (write in___________________) | 11 |
| None of these | 12 |

**ASK ALL**

**43. If a woman decides to or is able to breastfeed, what do you think is a good length of time to breastfeed for? DO NOT PROMPT, CODE ANSWER INTO RESPONSES BELOW, WRITE IN NUMBER OF MONTHS OR YEARS, MULTICODE POSSIBLE**

1. _____ Enter number of days
2. _____Enter number of months
3. ­_____ Enter number of years
4. Until she returns to work
5. For as long as possible
6. For as long as mother and baby are happy
7. Babies don’t need to be breastfed
8. I am not sure
9. Other ___________

**ASK ALL**

**44a Have you heard of ‘maternal milk’?**  **SINGLE CODE**

1. Yes **ASK 44C**
2. No

**ASK IF CODES YES TO 44a**

**44b Have you received any free samples of maternal milk?**

1. Yes
2. No

**44c Have you used any brand of ‘maternal milk’?** **MULTI CODE**

1. Yes, I tried it once, but didn’t continue
2. Yes, I am currently using it
3. Yes, I used it in a previous pregnancy
4. No, I have never tried it

**45 What do you think are the benefits, if any of breastfeeding a baby? DO NOT PROMPT, WRITE ANSWERS IN FULL**

1. ………………………...Open ended
2. None
3. Don’t know

**ATTITUDES TOWARDS INFANT FEEDING PRACTICES**

**ASK ALL**

**46. INTERVIEWER READ OUT: In the next question, we want to explore your attitudes towards feeding your baby. We are very interested in your opinions and experiences, there are no right or wrong answers.**

**[Note for interviewer: Do not show tablet]**

**Please can you say whether you agree, disagree or neither agree nor disagree with each statement that I am going to read out. SINGLE CODE FOR EACH, READ OUT EACH STATEMENT, ROTATE START POINT**

|  | **1**  **Disagree** | **2**  **Neither agree nor disagree** | **3**  **Agree** | **4**  **N/A** |
| --- | --- | --- | --- | --- |
| 1. Formula feeding is the better choice if the mother plans to go back to work | 1 | 2 | 3 | 4 |
| 1. Breastfeeding is best for your baby | 1 | 2 | 3 | 4 |
| 1. Formula fed babies grow better than breastfed babies | 1 | 2 | 3 | 4 |
| 1. Breastfeeding and formula feeding provide a baby with the same health benefits | 1 | 2 | 3 | 4 |
| 1. Formula helps babies sleep better | 1 | 2 | 3 | 4 |
| 1. Formula is very like breast milk | 1 | 2 | 3 | 4 |
| 1. Breastfeeding encourages better mother-baby bonding | 1 | 2 | 3 | 4 |
| 1. Formula keeps babies fuller for longer | 1 | 2 | 3 | 4 |
| 1. Breastfeeding in public is embarrassing | 1 | 2 | 3 | 4 |
| 1. Breastfed babies are healthier than formula fed babies | 1 | 2 | 3 | 4 |
| 1. Formula feeding allows you to get your life back quicker | 1 | 2 | 3 | 4 |
| 1. Breastfeeding is traditional | 1 | 2 | 3 | 4 |
| 1. Breastfeeding helps you get your body shape back quicker | 1 | 2 | 3 | 4 |
| 1. Men don’t like women breastfeeding | 1 | 2 | 3 | 4 |
| 1. You shouldn’t feel pressurised to breastfeed | 1 | 2 | 3 | 4 |
| 1. Formula feeding makes it easier to share feeding with your partner | 1 | 2 | 3 | 4 |
| 1. There should be much more support to help women breastfeed successfully | 1 | 2 | 3 | 4 |

**ASK ALL**

**INTERVIEWER READ OUT: ‘The next question explores how you feel about being or becoming a mother.**

**47. Please rate on a scale of one to five, with one being strongly disagree and five strongly agree, how you feel about the following statements: READ OUT STATEMENTS, SINGLE CODE FOR EACH**

|  | **1**  **Strongly disagree** | **2**  **Disagree** | **3**  **Neither agree nor disagree** | **4**  **Agree** | **5**  **Strongly agree** | **99**  **Not applicable** |
| --- | --- | --- | --- | --- | --- | --- |
| 1a. I am really enjoying being a mother **(MOTHERS)** | 1 | 2 | 3 | 4 | 5 | 6 |
| 1b. I am really looking forward to becoming a mother **(PREGNANT)** | 1 | 2 | 3 | 4 | 5 | 6 |
| 2. I am well supported by my friends and family | 1 | 2 | 3 | 4 | 5 | 6 |
| 3. I feel well supported by healthcare professionals | 1 | 2 | 3 | 4 | 5 | 6 |

**ADVERTISING AND MARKETING**

**ASK ALL**

**48. In the past year have you seen or heard any advertising for formula milk?** **When I say ‘advertising’, this could include information or promotions on formula milk, YouTube or other videos, sponsored websites, Facebook or other social media sites and chat rooms as well as TV, radio, billboard advertising etc. SINGLE CODE**

1. Yes
2. No
3. Don’t Know

**ASK IF CODES YES AT Q48, DO NOT PROMPT, OTHERS GO TO Q53**

**49. Thinking about the last advert that you saw or heard for formula milk, can you describe what the advert said? SAY ‘ANYTHING ELSE’**

Write in______________________________________________________________________________

**___________________________________________________________________________________**

**ASK IF CODES YES AT Q48**

**50. And where did you see or hear this advert? MULTICODE POSSIBLE**

1. TV
2. Radio
3. YouTube
4. Producer website e.g. a specific brand website (write in name ___________)
5. Professional or expert websites e.g. health information sites (write in name____________)
6. (write in name____________)
7. Social media e.g. Facebook, Instagram…
8. A health centre
9. A hospital
10. Magazine
11. Newspaper
12. Billboard e.g. on the highway or roadside
13. Supermarket/shop/market
14. In an elevator
15. Other (please write in _________________)

**ASK IF CODES YES AT Q48,**

**51. And do you remember what brand the advertising was for? DO NOT PROMPT, CODE BELOW OR WRITE IN**

1. Alpha
2. Aptamil
3. Dielac
4. Dumex
5. Dutch lady/Bella Holandesa
6. Enfagrow
7. Enfamil
8. Ensure
9. Friso
10. Grow
11. Lactogen
12. Meiji
13. Nan
14. Nutifood
15. Pediasure
16. Similac
17. Colosmama
18. Blackmores
19. Other (write in _______________________________)
20. Don’t know

**ASK IF CODES YES AT Q48**

**52. What type of formula milk was this advertising for: MULTICODE**

1. Infant formula
2. Follow-on formula
3. Toddler formula
4. Growing up milk
5. Stage 1 formula
6. Stage 2 formula
7. Stage 3 formula
8. Stage 4 formula
9. Milk to help babies grow
10. Other specialised milks? e.g. for sleep/ colic
11. Don’t remember/ Not sure

**ASK ALL**

**53. In the past year have you seen or heard any advertising about breastfeeding? When we say ‘advertising’, this could include information on breastfeeding videos, sponsored websites, social media content and chat rooms as well as TV, radio, billboard advertising etc. SINGLE CODE**

1. Yes
2. No
3. Don’t know

**ASK IF ANSWERS YES AT Q53, OTHERS GO TO Q56**

**54. Thinking about the last advert that you saw or heard about breastfeeding, can you recall what the advert said? SAY ‘ANYTHING ELSE’** Write in______________________________________________________________________________

**___________________________________________________________________________________**

**ASK IF ANSWERS YES AT Q53**

**55. Where did you see or hear this advert?**

1. TV
2. Radio
3. YouTube
4. Company website e.g. a specific brand website (write in name ___________)
5. Professional or expert websites e.g. health information sites (write in name____________)
6. Social media e.g. Facebook, Instagram…
7. A health centre
8. A hospital
9. Magazine
10. Newspaper
11. Billboard e.g. on the highway or roadside
12. Supermarket/shop/market
13. In an elevator
14. Other (please write in _________________)

**ASK ALL**

**56. Please tell me, have you ever received any of the following**

**INTERVIEWER, READ OUT EACH TYPE OF PROMOTION. SINGLE CODE FOR EACH. ROTATE START**

|  | **1**  **Yes** | **2**  **No** | **3**  **N/A** |
| --- | --- | --- | --- |
| 1. Information from a formula company by email, post, phone, or text message | 1 | 2 | 3 |
| 2. Promotion for formula discount | 1 | 2 | 3 |
| 3. Free samples of formula milk in hospital | 1 | 2 | 3 |
| 4. Free sample of formula milk outside hospital | 1 | 2 | 3 |
| 5. Free bottles or teats | 1 | 2 | 3 |
| 6. Any other gifts from a formula company e.g. a toy, bag or clothing | 1 | 2 | 3 |
| 7. An invite to join a ‘baby club’ run by a formula company | 1 | 2 | 3 |
| 8. An invite to a competition from a formula company or from a shop | 1 | 2 | 3 |
| 9. A pop-up advert on websites or Facebook or other social media | 1 | 2 | 3 |
| 10. Information about a helpline run by a formula company for example giving advice on pregnancy, birth or caring for your baby | 1 | 2 | 3 |
| 11. Private messages from formula companies on Facebook or other social media | 1 | 2 | 3 |
| 12. An invite from a formula company to participate in research/ survey/ interview | 1 | 2 | 3 |
| 13.Free samples of ‘maternal milk’ from a hospital or clinic | 1 | 2 | 2 |
| 14. Free samples of ‘maternal milk’ outside of hospital | 1 | 2 | 3 |
| 15. Consultation from a producer on new products | 1 | 2 | 3 |

**ASK ALL**

**57. And do you do any of the following? CODE AS MANY AS APPLY, MULTICODE**

1. Follow a formula company on social media
2. Use an app from a formula company
3. Participate in any baby competitions run by formula companies
4. Use any information from formula companies
5. Register for updates from a formula company
6. Register for a baby club run by a formula company
7. None of these above

**ASK ALL**

**58. INTERVIEWER READ OUT ‘Thinking of all the adverts that you have seen, can you remember whether any of these adverts featured any of the following messages SHOW SCREEN, CODE THOSE EVER SEEN. ROTATE ORDER**

**ASK ALL SHOW ALL MESSAGES ON SCREEN**

**59. And if you were looking to choose a brand of formula, which three of these messages would be most appealing to you? SINGLE CODE FOR EACH**

|  | **Q58. MESSAGES SEEN** | **Q59 1ST MOST APPEALING** | **Q59 2^ND^ MOST APPEALING** | **Q59 3^RD^ MOST APPEALING** |
| --- | --- | --- | --- | --- |
| 1. Improves babies sleep | 1 | 1 | 1 | 1 |
| 2. Improves babies health | 2 | 2 | 2 | 2 |
| 3. Improves babies brain development | 3 | 3 | 3 | 3 |
| 4. Promotes healthy weight gain | 4 | 4 | 4 | 4 |
| 5. Being similar to breastmilk | 5 | 5 | 5 | 5 |
| 6. Developed by scientists | 6 | 6 | 6 | 6 |
| 7. Easy to digest | 7 | 7 | 7 | 7 |
| 8. Reduces allergies | 8 | 8 | 8 | 8 |
| 9. Reduces relux | 9 | 9 | 9 | 9 |
| 10. Keeps babies fuller for longer | 10 | 10 | 10 | 10 |
| 11. Beneficial for toddlers | 11 | 11 | 11 | 11 |
| 12. Beneficial for 6 months plus | 12 | 12 | 12 | 12 |
| 13. Breastmilk is best | 13 | 13 | 13 | 13 |
| 14.Helps babies/children grow taller | 14 | 14 | 14 | 14 |
| 15. Increases immunity to illnesses | 15 | 15 | 15 | 15 |
| 16.Contains essential nutrients | 16 | 16 | 16 | 16 |
| 17.Contains HMO | 17 | 17 | 17 | 17 |
| 18.Contains DHA | 18 | 18 | 18 | 18 |

**ASK ALL INTERVIEWER PLEASE READ OUT:**

**60. I am now going to read out some statements about advertising for formula milk, for each, please can you say whether you agree or disagree or neither agree not disagree. SINGLE CODE, ROTATE ORDER OF READING**

|  | **Disagree** | **Neither agree nor disagree** | **Agree** | **Don’t know** |
| --- | --- | --- | --- | --- |
| 1. Formula ads helped me to make decisions | 1 | 2 | 3 | 4 |
| 2. I think that the ads are accurate | 1 | 2 | 3 | 4 |
| 3. I don’t think formula companies should be allowed to promote their products | 1 | 2 | 3 | 4 |
| 4. Formula ads discourage mothers from breastfeeding | 1 | 2 | 3 | 4 |
| 5. I feel reassured by the information they provide | 1 | 2 | 3 | 4 |
| 6. They are no different to ads for other products | 1 | 2 | 3 | 4 |
| 7. I feel like formula milk advertising is everywhere | 1 | 2 | 3 | 4 |

**INTERVIEWER -** **SHOW COLLAGE OF EXAMPLE ADVERT IMAGES**

**ASK ALL**

**61. ‘We are now going to show you some examples of advertising for formula milks. On the screen you will see a list of places where you may have seen this type or similar advertising for formula brands.**

**Firstly, please can you say in which, if any of these locations, you have ever seen any of this type of advertising? CODE AS MANY AS APPLY, MULTI CODE**

**SHOW THOSE LOCATIONS MENTIONED AT 61 ONLY**

**62. And in which of these locations have you frequently seen this type or similar advertising?**

**CODE AS MANY AS APPLY, MULTI CODE**

|  | **Q61 Ever seen ads here** | **Q62 Frequently seen ads here** |
| --- | --- | --- |
| 1. In a hospital or clinic | 1 | 1 |
| 2. In a pharmacy | 2 | 2 |
| 3. In a magazine or newspaper | 3 | 3 |
| 4. On the television | 4 | 4 |
| 5. In the street e.g. a billboard | 5 | 5 |
| 6. In a shop | 6 | 6 |
| 7. In the post | 7 | 7 |
| 8. In an email | 8 | 8 |
| 9. In an advert by a formula company on Facebook or other social media | 9 | 9 |
| 10.In an advert from a shop on Facebook | 10 | 10 |
| 11. On a social media account that I follow e.g. celebrity, mummy blogger | 11 | 11 |
| 12. On an online discussion forum | 12 | 12 |
| 13. On another website | 13 | 13 |
| 14. In a competition | 14 | 14 |
| 15. In a mother’s club | 15 | 15 |
| 16.Other- please write in  ____________________________ | 16 | 16 |

**ASK ALL WHO USE/OR WILL USE FORMULA (Screener Q5/ 2,3,4 or 5) (Screener Q7/ 2,3,4,5)**

**63. Does/Will feeding your baby formula significantly impact on your family’s finances?**

1. Yes
2. No

**ASK ALL, SHOW IMAGES OF FORMULA MILK PROMOTIONS**

**64a. When you are shopping for formula milk, either in a store or online, which of the following types of promotions and discounts have you ever seen? CODE ALL MENTIONED, FOR EACH CODE WHETHER ONLINE OR IN STORE**

**ONLY SHOW THOSE CODED AT 64a**

**64b. Which of these types of promotions and discounts have you ever taken part in / used, either in store or online? CODE ALL MENTIONED, FOR EACH CODE WHETHER ONLINE OR IN STORE**

|  | **Q64a Ever seen in store** | **Q64a Ever seen online** | **Q64b Ever seen in store** | **Q64b Ever seen online** |
| --- | --- | --- | --- | --- |
| **Points collection redeemed as gifts, when buying formula** | **1** | **1** | **1** | **1** |
| **Discounted price for formula** | **2** | **2** | **2** | **2** |
| **Multi-buy discount e.g. Buy 2 packs get the third free** | **3** | **3** | **3** | **3** |
| **Lucky draws/ competitions to win prizes** | **4** | **4** | **4** | **4** |
| **Free gift with purchase of formula** | **5** | **5** | **5** | **5** |
| **Other write in**  **----------------**  **­­­­­­­­­­­­_________________** | **6** | **6** | **6** | **6** |

**THANK YOU VERY MUCH FOR TAKING PART IN THIS SURVEY**

**END OF SURVEY**

**INTERVIEWER - READ THE FOLLOWING**

*Thank you for your time, your responses are very much appreciated.*

*As stated previously your data will be stored securely. Your anonymised data will be shared with the World Health Organization for public health research purposes, and WHO may authorise, during long term storage, third party use of data for purposes relevant to the subject of the data.*

*Can you please confirm that you are happy for your data to be shared with the World Health Organization?*

**INTERVIEWER - COLLECT VERBAL CONSENT**

**Does this participant give verbal consent?**

1. **Yes**
2. **No - IF THE PARTICIPANT SAYS NO THEN THE INTERVIEW IS NULL AND VOID**

If you have any questions or concerns about how to feed or care for your baby, please contact your health professional or the following regional services:

**[INSERT LOCAL SUPPORT SERVICES CONTACT INFORMATION]**

Please direct any further research enquiries to the research team:

**[INSERT LOCAL AGENCY CONTACT INFORMATION]**
