# Supplementary material for: Women’s exposure to commercial milk formula marketing: a WHO multi-country market research study
Source: Global Health. 2024 Nov 28;20:85. doi: 10.1186/s12992-024-01088-y (PMC11603767; doi:10.1186/s12992-024-01088-y)
Supplement: Supplementary file 4 — Supplementary Material 4. [file 12992_2024_1088_MOESM4_ESM.docx]

**Appendix 4: Household Assets used to determine socioeconomic status in Nigeria, South Africa and Mexico**

Household asset data collected in Nigeria were as follows

- Household help
- Fridge / deep freezer
- Video
- Car
- Colour TV
- Music system
- Air conditioning unit
- Satellite dish
- Washing machine
- Black and white TV
- DVD player
- Telephone (land)
- Telephone (mobile)
- Personal driver
- Multiple cars
- Household income

Household asset data collected in South Africa were as follows

- Built-in kitchen sink
- Tap water inside your home or store-bought water for use in your home
- Hot running water from a geyser
- Flush toilet inside the house
- Home security service
- Motor car
- Fridge or combined fridge/freezer
- Deep freezer that is free standing
- Microwave oven
- Floor polisher or vacuum cleaner
- Washing machine
- I use taxis for transport
- I use trains for transport
- I watch free to air TV
- I watch open view TV
- I watch paid to view TV
- Household income.

Household assets data collected in Viet Nam were as follows

- Laptop computer
- Bank account
- Mattress
- Refrigerator
- Television
- Motor bike
- Mobile with email facility / smart phone
- Air conditioning (built in NOT portable)
- Dining table
- Motor car
- Household income.
